# Epidural Catheter Migration: A Case Report of a CT Scan Examination

**DOI:** 10.7759/cureus.30831

**Published:** 2022-10-29

**Authors:** André Eloy, Joana Tinoco, Rita Regufe, Jorge Cortez, Lisbete Cordeiro

**Affiliations:** 1 Anesthesiology Department, Centro Hospitalar de Setúbal, Setúbal, PRT

**Keywords:** epidural analgesia, epidural anesthesia, epidural catheter complication, epidural catheter displacement, epidural catheter migration, paravertebral space, psoas muscle

## Abstract

Epidural catheter placement is one of the most effective, secure, and worldwide used pain control modalities. Epidural catheter dislodgment is a common cause of epidural block failure. The diagnosis of this situation is usually presumptive, and cases in which the actual trajectory and final location of the catheter are witnessed by imaging are rare.

We present two cases of the insufficient epidural block due to catheter migration, confirmed by a CT scan with radiopaque contrast injection through the catheter. In the first case, the catheter tip was identified in the left major psoas muscle. Some catheter holes were probably located in a border zone between two compartments, which made the analgesic efficacy dependent on the infusion rate. In the second case, the catheter tip was identified as lodged in the left paravertebral space, which explains only unilateral left pain relief.

In selected situations, like repeated ineffectiveness and in pretended long-duration catheters, imaging tests may be useful to determine the actual position of the catheter and identify anatomical variations that may lead to an incorrect replacement.

## Introduction

Epidural catheter placement is one of the most effective ways to obtain analgesia for surgical procedures, labor pain, and chronic pain syndromes. Despite this, it tends to present a failure rate of around 23% [1]. Epidural block can fail due to incorrect initial positioning, correct initial positioning with subsequent displacement, or an insufficient dose of local anesthetic [2]. 

The diagnosis is usually presumptive and resolved with a 2 cm withdrawal or replacement of the catheter. Probably, in specific situations, imaging studies should be performed to identify the real cause of the ineffectiveness of epidural block and provide orientation for the placement of a new catheter. To attest to the real location of the catheter after placement, some carried out epidurography [3].

Some studies used the comparison between different epidural catheter placement depths [4,5]. A depth of 5 cm of the epidural catheter seems to be the most appropriate length for postoperative analgesia, with minimum catheter-related complications such as dislodgment and unilateral analgesia more frequently found in the 3 or 7 cm length groups [3], respectively.

With imaging, it was found that in a large number of cases, the catheter was not in the presumed location [6,7]. Even after correct identification of the epidural space, the catheter may follow an erroneous path as it is advanced, entering and exiting the epidural space, and may be installed in structures above or below the placement site. The anatomy of the peri-epidural space is important. Near the lumbar spine and the epidural space, several structures can be reached by the epidural catheter in case of migration, such as the immediately adjacent paravertebral space [8] and the musculature of the lumbar region [9], for example, major psoas or quadratus lumborum muscles.

We present two cases of the insufficient analgesic block due to migration of the epidural catheter in patients who were followed in chronic pain consultation. We believe that this type of case report is important to demonstrate the feasibility of performing CT scan contrast epidurography. It is a useful approach in patients with ineffective analgesia through the epidural catheter, and in specific situations, it constitutes better patient care.

## Case presentation

Clinical case 1

A 42-year-old woman was referred to the chronic pain unit (CPU) by locally advanced cervical cancer, Federation of Gynecology and Obstetrics (FIGO) stage IIB [10], with constant pain in the right hypochondrium and suprapubic region. She was medicated with paracetamol, ibuprofen, clomipramine, diazepam, quetiapine and paroxetine. After pain team consultation, analgesic therapy optimization was attempted with metamizole, pregabalin, amitriptyline, and transmucosal fentanyl as a rescue. The patient did not have consistent pain relief and was therefore proposed neuraxial epidural analgesia. An epidural set (B.Braun Perifix® filter set) composed of an 18G Tuohy needle and 1000 mm length G20 catheter with triple-drilled soft tip and multilateral holes was used. The procedure was performed by a senior anesthesiologist with over 30 years of experience in chronic pain management. In the right lateral decubitus position, using the loss of resistance technique with saline, an epidural catheter was placed at the L3-L4 level with a 4 cm introduction into the epidural space. The catheter was tunneled through the skin, and 5 mg morphine plus 40 mg methylprednisolone in 10 mL saline were incrementally administered, with complete pain relief after 10 minutes of the last administration, without sensory or motor deficits. The patient was discharged home with a 300 mL drug infusion balloon (DIB) with 30 mg morphine and 300 mg ropivacaine to be infused epidurally at a rate of 5 ml/h.

On the second day after placement, the patient returned to the CPU due to a recurrence of severe pain, requiring rescue analgesia. The catheter was permeable and not externalized. A bolus of 8 ml ropivacaine 0.2% (16 mg) and 2 mg morphine was administered, in 10 ml, with pain relief. It was decided to double the dose of morphine in the DIB, maintaining the same infusion rate. She came back eight, 11, and 17 days later due to severe pelvic pain, keeping the catheter functioning and in the same depth, with morphine concentration being progressively increased to a maximum of 120 mg and ropivacaine to 650 mg at a fixed infusion rate.

**Figure 1:**
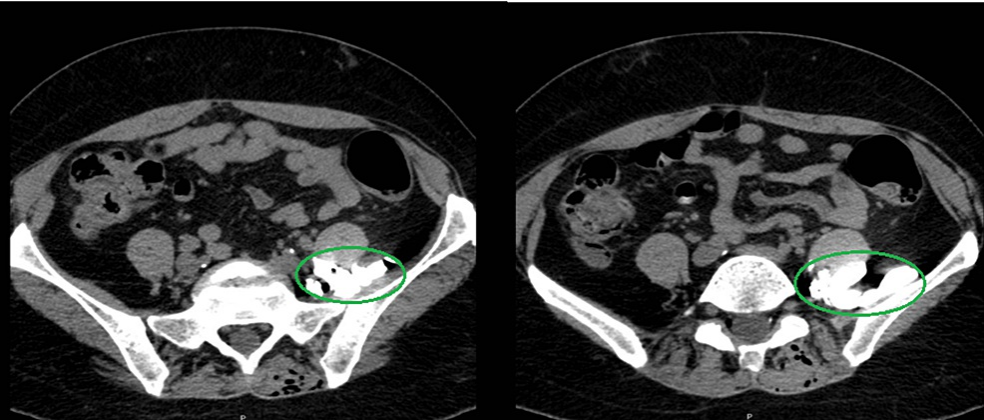
Transverse CT scan after administration of radiopaque contrast through the epidural catheter in case 1 Circles show enhancement in the left psoas major muscle after epidural contrast administration.

It was decided to replace the catheter with a new one. The catheter was placed by the same clinician in the L3-L4 intervertebral space using the same technique and introduced 4 cm in the epidural space in the caudal direction, with pain relief after 10 minutes of a test dose. Less than 24 hours later patient reports a highly intense recurrence of pain. It was then hypothesized that the catheter tip was not in the epidural space. Radiopaque contrast was administered through the catheter, and an abdominopelvic CT scan was performed. The left major psoas muscle captured contrast, and it was possible to visualize the catheter tip exiting through the homolateral intervertebral hole (Figure 1). The catheter was then removed and replaced with a new one under fluoroscopy guidance and patient sedation. It took several attempts at different levels to correctly place it, with radiological control showing the adoption of an erroneous trajectory of the catheter tip.

Clinical case 2

A 68-year-old woman was referred to CPU with unresectable pancreatic cancer, the American Joint Committee on Cancer (AJCC) stage IV [11], liver metastases, and celiac plexus involvement with constant low back, epigastric and bilateral flank pain and a history of scoliotic and osteophytic processes of the lumbar and dorsal spine. She was medicated with metamizole, tramadol, etodolac, and tizanidine. After pain consultation, analgesic optimization was attempted with morphine (up to 30 mg), but due to the adverse effects and rapidly progressive tendency of this type of cancer, placement of an epidural catheter was proposed. 

In the right lateral decubitus position, using the loss of resistance technique with saline, the same epidural catheter set (B.Braun Perifix® filter set) was placed by the same experienced clinician of the chronic pain department at the L2-L3 level with a 4 cm introduction into the epidural space, ideally reaching T12 level. The catheter was tunneled through the skin and tested with 100 mcg fentanyl and 2 ml ropivacaine 0.2% (4 mg) administration with good pain relief after 15 minutes and no neurological deficits. She was discharged home with an epidural DIB with 600 mcg fentanyl and 400 mg ropivacaine in a total volume of 300 mL, at a rate of 5.2 ml/h and an indication to stop oral opioids and reduce the daily dose of etodolac. 

**Figure 2:**
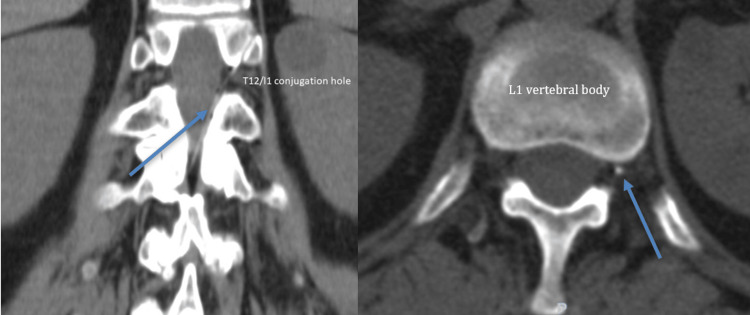
Coronal and transverse CT scan after administration of radiopaque contrast through the epidural catheter in case 2 The left arrow shows the catheter pathway inside the epidural space with ascending and left anterior trajectory and exiting through the T12/L1 conjugation hole. The right arrow shows the approximate final position of the catheter tip at L1 level left paravertebral space.

The patient returned on the second day after catheter placement due to a gradual worsening of pain complaints, with only slight relief on the left flank. She was hospitalized and medicated with transdermal fentanyl 25 mcg and obtained some pain relief with adverse symptoms such as nausea and constipation. Because the catheter was permeable and not externalized, it was decided to double the concentration of fentanyl and raise the ropivacaine dose in the DIB to 600 mg with the same infusion rate, obtaining only partial relief. On the following day, the dose of fentanyl and ropivacaine in the DIB increased again with the maintenance of severe pain complaints and requiring the use of intravenous morphine. Then, on the fifth day of hospitalization, the patient underwent a CT scan of the dorsal and lumbar spine with radiopaque contrast injection through the catheter. It was possible to visualize the catheter entering the epidural space at the L2-L3 level with an ascending and left anterior path and exiting through the homolateral D12-L1 conjugation hole, staying lodged in the left paravertebral space (Figure 2). Thus, it was decided to externalize the catheter 2 cm and resume the initial infusion dose through the DIB with complete and lasting pain relief.

## Discussion

The relevance of these clinical cases correlates with the importance of epidural analgesia in chronic pain situations, especially oncological. The lack of widely accessible methods for confirming objective success has contributed to failure rates, partially limiting clinical efficacy. Despite some rate of ineffectiveness, this continues to be an appropriate tool in controlling severe cancer pain, mainly due to its low rate of complications and adverse effects but also for its opioid-sparing capabilities [12]. 

Epidural block failure can be related to several factors, such as anatomical alterations of the spine, poor positioning of the patient, needle type, insertion site (median or paramedian), type of technique used to locate the epidural space, and, not least important, excessive depth of the catheter that favors migration and externalization [2]. In most situations, without the use of imaging methods, it is not possible to understand the cause of epidural block failure, and in literature, the image documentation of cases of aberrant placement is rare. 

Two cases of catheter migration to the major psoas muscle were described in the literature. One reports the absence of an analgesic block through the epidural catheter placed in a sequential technique, after subarachnoid anesthesia, for foot amputation surgery. As shown, the contrast was injected via the catheter, and a CT scan was performed, which revealed contrast uptake in the psoas major muscle [13]. The other report [14] affirms direct visualization of the catheter tip in the psoas muscle during radical nephrectomy of a preoperatively placed lumbar epidural catheter. In the first report [13], the authors highlighted the fact that there is effective analgesia when the bolus is administered, and analgesia failure only manifests on continuous fixed-rate infusion with DIB. That was the reason why the hypothesis of catheter migration was not initially raised. In our first case, although there is no image confirmation of the placement of the first catheter, we believe that a plausible explanation for what happened is that the distal portion of the catheter was at the level of the intervertebral hole. Today’s most commonly used catheters are triple-drilled and, as such, have three possible exit sites. Several studies report that there are a significant number of cases in which the three holes stay placed in two adjacent compartments [15,16]. The clinical manifestation of this phenomenon appears to be infusion rate dependence. The selective obstruction of one orifice and the existence of anatomical alterations that favor the adoption of an aberrant path, as seen in our fluoroscopy-guided placement, may have also contributed. Even under fluoroscopic guidance, it was not easy to replace the catheter. We tried a different level approach than L3-L4, where previous non-fluoroscopic placements were made. Pain relief was possible only after a correct and final placement on our third attempt in the L2-L3 level. In addition to the different epidural spaces attempted, there are other limitations when performing this technique under fluoroscopy guidance in the operating room that may have contributed. Namely, space limitations, space conflict with the fluoroscopic device, and the difficult positioning of a non-collaborative patient under sedation.

Literature also describes some cases of inadvertent paravertebral placement of thoracic [17] and lumbar [16] epidural catheters. These were discovered by direct visualization during surgery. Due to the proximity of the two spaces, the inadvertent epidural placement of a paravertebral block catheter has also been reported [18]. This paravertebral location may explain the unilateral partial pain relief in the left flank manifested by our second case patient.

The position of the catheter tip and distribution of injected solution into the epidural space show extraordinary variability between patients. Several studies and case reports [16,19,20] proposed to investigate the progression of the catheter in the epidural space and the final position of the catheter tip. The idea is to better understand this variability and, at the same time, demonstrate the feasibility and safety of these methods of epidural contrast injection in patients undergoing a CT scan. It is especially important for identifying dangerous locations of the tip but also of the catheter orifices, e.g., intravascularly, within organs, or in paravertebral musculature, where it can cause local anesthetic myotoxicity, muscle spasm, and increase in pain complaints. 

With the clinical cases presented here, we intend to demonstrate the usability, indication and benefit that CT scan epidurography and fluoroscopy-guided epidural catheter placement can have in some patient situations. Although it seems that epidural contrast injection can be performed without any deleterious consequences [20], there must be some criteria for performing CT scan contrast epidurography and not performing it in all patients with a dysfunctional catheter. This technique should be reserved for patients with ineffective catheters that are pretended to be for long duration or in which epidural analgesia is undoubtedly the most advantageous analgesic technique for the patient. Epidurography CT scan and then a fluoroscopic epidural catheter placement should be a good option for catheters that have already been replaced more than once and remain non-functioning or when the dysfunction of the catheter is supposedly caused by an anatomical variation affecting the epidural space. Diagnosing catheter migration with imaging methods can be especially useful in altered and difficult anatomies in order to understand the variations beyond the catheter displacement. It also gives orientation to correctly place a new catheter, increasing the effectiveness of the procedure and reducing the number of complications.

Patients followed in pain consultation for chronic pain, especially oncological, seem to be the ones who benefit the most from this type of procedure since, in acute situations, we can easily complement analgesia with other multimodal techniques. In these oncological chronic pain patients, a long-term epidural catheter is often the last option available in many hospital centers.

Like any medical procedure, epidural space imaging with contrast administration can have some negative side effects. Nevertheless, they seem to occur with less frequency than when contrast is administered in other spaces, like direct intravenous administration, either because of the smaller amount and concentration used to visualize the epidural space or because of the lower systemic distribution that occurs when compared to intravenous administrations.

Side effects of radiographic contrast media can range from mild inconveniences, such as itching and cutaneous reactions, to life-threatening emergencies like anaphylactic reactions. We anticipated the risk of anaphylactic reactions and delayed adverse effects such as renal impairment and thyroid or hepatic dysfunction that none of our patients experienced nor the ones in the existent studies and case reports [16,19,20].

Although the risk of adverse effects from epidural contrast administration appears to be low, the decision to perform this type of procedure should take into account the risk-benefit assessment of each patient situation. 

Because there is no established method for this procedure, we believe that we should choose to inject the lowest dose in the minimal concentration to be safe and effective. In some cases, especially in young and healthy individuals with a normal life expectancy, it is preferable not to evaluate the longitudinal spread of the solution to limit radiation exposure.

## Conclusions

When desired analgesic efficacy is obtained after epidural drug administration, it is assumed that the catheter is functioning and, therefore, well located. However, good initial functioning of the catheter does not exclude its posterior migration to undesired locations and consequent loss of analgesic efficacy. The study of the position of the catheter using an injection of contrast and imaging methods assumes not only academic importance but better patient care. As we have seen, intermittent ineffectiveness with boluses may indicate a situation of partial migration of the catheter tip from the epidural space with some catheter holes still inside the space. In situations of repeated ineffectiveness, it may be important to perform imaging tests to determine the actual position of the catheter and identify anatomical variations that may lead to a new incorrect placement, providing indications on the best approach for the replacement of the catheter. 

Epidurography is a safe radiographic contrast study that can provide immediate confirmation of the epidural catheter location and eliminate mispositioned catheters that can be in an ineffective or dangerous location. As with any medical procedure, it should only be performed in a specific group of patients and situations, always taking into account the risk-benefit assessment of the situation.
